# Social media use and sleep health among adolescents in Canada

**DOI:** 10.24095/hpcdp.44.7/8.05

**Published:** 2024-07

**Authors:** Florence Lafontaine-Poissant, Justin J. Lang, Britt McKinnon, Isabelle Simard, Karen C. Roberts, Suzy L. Wong, Jean-Philippe Chaput, Ian Janssen, Meyran Boniel-Nissim, Genevive Garipy

**Affiliations:** 1 School of Public Health, University of Montreal, Montral, Quebec, Canada; 2 Quebec Region, Regional Operations, Public Health Agency of Canada, Montral, Quebec, Canada; 3 Centre for Surveillance and Applied Research, Public Health Agency of Canada, Ottawa, Ontario, Canada; 4 School of Epidemiology and Public Health, Faculty of Medicine, University of Ottawa, Ottawa, Ontario, Canada; 5 Healthy Active Living and Obesity Research Group, Children’s Hospital of Eastern Ontario Research Institute, Ottawa, Ontario, Canada; 6 Dalla Lana School of Public Health, University of Toronto, Toronto, Ontario, Canada; 7 Centre for Health Promotion, Public Health Agency of Canada, Ottawa, Ontario, Canada; 8 Department of Pediatrics, Faculty of Medicine, University of Ottawa, Ottawa, Ontario, Canada; 9 School of Kinesiology and Health Studies, Queen’s University, Kingston, Ontario, Canada; 10 Department of Public Health Sciences, Queen’s University, Kingston, Ontario, Canada; 11 Department of Educational Counseling, The Max Stern Academic College of Emek Yezreel, Israel

**Keywords:** adolescents, social media use, sleep, insomnia, daytime wakefulness, sleep duration, sleep variability

## Abstract

**Introduction:**

Public health concerns over the impact of social media use (SMU) on adolescent health are growing. We investigated the relationship between SMU and sleep health in adolescents in Canada aged 11 to 17 years.

**Methods:**

Data from the 2017–2018 Health Behaviour in School-aged Children study were available for 12557 participants (55.2% female). SMU was categorized by frequency of use (non-active, active and intense) and the presence of addiction-like symptoms (problematic). Mixed effects logistic regression models identified associations between SMU and seven sleep health indicators (insomnia symptoms, daytime wakefulness problems, screen time before bed, meeting sleep duration recommendations, sleep variability and late bedtime on school and non-school days).

**Results:**

Compared to active SMU, non-active SMU was associated with better sleep indicators, except for insomnia symptoms. Intense SMU was associated with greater odds of having poor sleep health indicators (adjusted odds ratio [aORs] from 1.09 to 2.24) and problematic SMU with the highest odds (aORs from 1.67 to 3.24). Associations with problematic SMU were greater among girls than boys, including having a later bedtime on school days (aOR=3.74 vs. 1.84) and on non-school days (aOR = 4.13 vs. 2.18). Associations between SMU and sleep outcomes did not differ by age group.

**Conclusion:**

Intense and problematic SMU were associated with greater odds of poor sleep health among adolescents in Canada, with stronger associations among girls than boys. Further research is needed to understand the mechanisms underlying associations between SMU and sleep to inform public health recommendations.

**Highlights**

- Intense and problematic social media use were both associated with worse sleep health compared to active social media use.
- The highest odds of having poor sleep health indicators were associated with problematic social media use (adjusted odds ratios from 1.67 to 3.24) assessed using the Social Media Disorder Scale.
- Non-active social media use was linked to better sleep health.
- Associations between poor sleep health indicators and social media use were stronger among girls than boys.
- Across social media use categories, odds ratios for having poor sleep health indicators did not differ by age group.

## Introduction

Social media use (SMU), defined as the time spent on social media platforms (e.g. Facebook, Twitter, TikTok, etc.) to connect with other users and exchange user-generated content, is an integral part of adolescents’ lives around the world.^1^,^2^ In the United States, the percentage of adolescents reporting intense SMU (i.e. being online almost constantly) increased from 25% to 45%.^3^ One cross-national study estimated that 40% of adolescents aged 15 to 19 years increased their SMU during the COVID-19 pandemic.^4^

In the current literature, a distinction is made between intense and problematic SMU. Intense SMU is defined as spending a lot of time on social media, whereas problematic SMU implicates the presence of behavioural and psychological symptoms of addiction that affect daily functions.^2^,^5^ While social media offers opportunities to strengthen friendships, promote social support and reduce social isolation, intense and problematic SMU may negatively impact youth health and well-being, including sleep.^2^,^5^,^6^

Sleep is essential to the health and development of adolescents and a contributor to their well-being through its influence on learning capacities, emotional regulation and memory processes.^7^ Sleep health encompasses not just sleep duration but also sleep quality, regularity, satisfaction, appropriate sleep timing alertness during the day, and sleep-facilitating behaviours.^8^,^9^ These components of sleep have been proposed in a sleep health framework called Peds B-SATED (Behaviour, Satisfaction/Quality, Alertness/Sleepiness, Timing, Efficiency and Duration).^9^ In Canada, one in three children and adolescents do not meet sleep duration recommendations,^10^,^11^ and at least 25% have symptoms of insomnia and daytime wakefulness problems^10^

Numerous studies have linked SMU with poor sleep health in adolescents.^12^,^13^ SMU is hypothesized to impact sleep via four mechanisms: (1) exposure to blue light, which affects circadian timing; (2) psychophysiological activation due to the emotional content of social media; (3) the “never-ending” nature of SMU; and (4)the constant alerts that disturb sleep.^2^,^6^,^14^ Studies linking SMU to sleep have generally focused on one aspect of sleep, most often sleep duration, but interest is growing in understanding the association between SMU and other aspects of adolescent sleep, such as sleep quality and sleep-facilitating behaviours.^8^,^9^,^15^ Yet, according to a 2019 census on SMU in children and youth aged 8 to 18 years in the United States, only 14% reported that their parent monitored their time spent on social media.^16^

Associations between SMU and sleep may vary by gender and age. A study of adolescents in the United States and the United Kingdom found that the association between time spent on social media and lower well-being was greater among girls than boys. 17 Another study also identified significant differences in SMU by age group, with 11-year-olds reporting less intense SMU than 13- and 15-year-olds and significantly better mental health across most measures,^2^ suggesting a need to look at age differences more closely. Studying gender and age differences in the associations between SMU and different sleep health indicators may offer a clearer picture of the factors in the relationship between SMU and sleep.

The aim of the current study is to investigate the association between SMU and sleep health indicators among adolescents in Canada and to examine any gender and age differences. We examined SMU with a previously developed scale^2^ that combines intensity and problematic symptoms. We hypothesized that intense and problematic SMU would be associated with worse sleep health, compared to active SMU, and that associations would be stronger in girls than boys and in older than younger adolescents.

## Methods

***Data and participants***

Data were from the 2017–2018 Health Behaviour in School-aged Children (HBSC) study, a cross-national research study and World Health Organization collaboration, that collects data every 4 years from a representative sample of students in Grades 6 to 10 in the school setting. The Canadian part of the survey used a random two-stage cluster sample of students from all provinces and two territories (Yukon and Northwest Territories). Data were collected between January and May of 2018. Participation was voluntary and anonymous. The Canadian HBSC study obtained student assent and active and/or passive parental consent, depending on school board requirements. 

The General Research Ethics Board at Queen’s University (GMISC-062-13) and the Health Canada–Public Health Agency of Canada Research Ethics Board provided ethics approval.

A total of 21745 students from 287 schools participated in the survey. For this study, we excluded adolescents in Grade 5 (n=40) and Grade 11 (n=163) because the HBSC survey is representative of students in Grades 6 to 10. We excluded adolescents who responded to “neither term describes me” for gender (n=325) because of the small sample size, and those with information missing on SMU (n=6226) and on the variables in our analyses (n=2434), resulting in a final sample of 12557 students with complete data.

***Measures***

**Social media use**

To assess SMU intensity, the survey asked participants to identify how often they had online contact with the following four categories of people: close friends; friends from a larger friend group; friends they met through the Internet; and other people (such as classmates, siblings or teachers). Response options were: “never or almost never,” “at least every week,” “daily or almost daily,” “several times each day” and “almost all the time throughout the day.” The highest frequency reported across the four categories was used to establish three levels of SMU intensity: (1)non-active (never or at most weekly); (2) active (daily/several times a day); and (3) intense (almost all the time), as previously described by Boniel-Nissim et al.^2^

We assessed problematic SMU using the Social Media Disorder Scale.^18^ The scale has previously demonstrated appropriate validity in a large international sample of adolescents.^19^ The scale includes nine “yes/no” items that identify addiction-like symptoms of SMU during the past year (e.g. having conflict with family, lying about the amount of time spent on social media, feeling bad when cannot use social media, among others). Participants who responded yes to six or more items were classified as problematic users, regardless of their SMU intensity level.^2^

Participants were classified into one of four mutually exclusive categories: non-active SMU (non-active SMU and nonproblematic use); active SMU (active SMU and nonproblematic use); intense SMU (intense SMU and nonproblematic use); and problematic SMU (problematic use regardless of SMU intensity).^2^

**Sleep health**

We investigated seven indicators of sleep health, based on availability in the dataset: insomnia symptoms, daytime wakefulness, screen time before bed, sleep duration, sleep variability, and sleep timing on school days and on non-school days (weekends and holidays). Many of these sleep health measures align with the Peds B-SATED framework, with four of the six domains included.^9^ We were unable to investigate sleep satisfaction/quality and efficiency with the current data source.

**Insomnia symptoms**

Participants were asked how often they have trouble going to sleep or staying asleep. There were five response options: “never,” “rarely,” “sometimes,” “most of the time” and “all the time.” The variable was dichotomized as those with insomnia symptoms (“most of the time” and “all the time”) and those without (“never,” rarely” and “sometimes”), in line with previous work.^20^

**Problems with daytime wakefulness**

Participants were asked how often they have trouble staying awake during the daytime when they want to be awake. There were five response options: “never,” “rarely,” “sometimes,” “most of the time” and “all the time.” “Never” and “rarely” were grouped to create a dichotomous variable defined as no daytime wakefulness problems.

**Screen time before bed**

To assess sleep-facilitating behaviours, the survey asked participants how often they watched television or used a cellphone or computer/tablet in their bedroom in the last hour before going to sleep. There were five responses options: “never,” “1or 2 nights a week,” “3 or 4 nights a week,” “5 or 6 nights a week” and “every night.” Participants who responded “never” or “1or 2 nights a week” were categorized as using screens before bed less than 2nights a week, and all the other participants were categorized as using screens before bed 3 or more nights a week.

**Sleep duration**

To assess sleep duration, the survey asked participants when they usually go to bed and when they usually wake up, on school days and non-school days (weekends and holidays), separately. Participants could answer within 15-minute increments. Sleep duration on school days and non-school days was calculated and used to determine average daily sleep duration, which was compared to sleep duration recommendations for adolescents. Sleep duration recommendations differ depending on age group: 9 to 11 hours per night for 11- to 13-year-olds and 8 to 10 hours per night for 14- to 17-year-olds.^21^ Taking into account sleep duration recommendations by age, we separated participants into two categories, meeting the sleep duration recommendation or not meeting the sleep duration recommendation.

**Sleep variability**

To assess sleep variability/regularity, we calculated for each participant if there was more than a 2-hour difference between bedtime during the week and weekend nights.^22^ Participants with less than a 2-hour difference between bedtime during the week and weekend nights were categorized as having little or no sleep variability.

**Sleep timing**

As wake times can largely depend on school start times, we used bedtimes as an indicator of sleep health. We calculated bedtime tertiles on school and non-school days for each age category (11–13 years and 14–17 years), given the shift towards a later bedtime during adolescence due to biological changes in circadian rhythms. We then categorized participants as having an early/moderate bedtime (first and second tertile) or a late bedtime (third tertile) on school days in comparison with peers. For those aged 11 to 13 years, late bedtimes on school and non-school days were after 10:30 p.m. and 12:00 a.m., respectively. For youth aged 14 to 17 years, late bedtimes on school and non-school days were after 12:00 a.m. and 1:00 a.m., respectively.

***Sociodemographic variables***

We included information on gender (boy/girl), cultural/ethnoracial background (categorized as White vs. non-White) and family affluence. Family affluence was measured using the Family Affluence Scale, a reliable and valid measure of socioeconomic status.^23^ The FAS is a composite score based on household characteristics, including the number of cars, bathrooms, computers, having an unshared bedroom and the number of family holidays abroad during the past year. The responses were summed and categorized into three groups (0–6: “low affluence”; 7–10: “medium affluence”; and 11–13: “high affluence”).^23^

***Statistical analysis***

We first conducted descriptive analyses of the sample across the four SMU categories. To examine the associations between the four SMU categories and sleep variables, we used mixed effects logistic regression models, with separate models for each of the seven sleep health outcomes. From the logistic regression models, we report both the odds ratio and 95% confidence interval (CI). All models were adjusted for gender, cultural/ethnoracial background, age and family affluence. 

We then conducted additional exploratory analyses to examine gender and age differences by rerunning the models stratified by gender and age group. All models were controlled for clustering by schools using mixed effects models, and survey weights were applied to ensure results were representative of Grade 6 to 10 students in Canada. An alpha value of 0.05 was used to detect statistically significant results.

We conducted analyses in SAS Enterprise Guide version 7.1 (SAS Institute Inc., Cary, NC, US). 

## Results

***Descriptive characteristics***

The most common SMU category was active users (43.7%) followed by intense users (35.4%), non-active users (14.2%) and problematic users (6.7%). Problematic and intense users were generally more likely to be girls and non-White compared with active users. Conversely, non-active users were generally more likely to be boys and younger than the active SMU groups (Table 1). 

**Table 1:** Descriptive characteristics of the sample overall and by social media use category (n = 12 557)

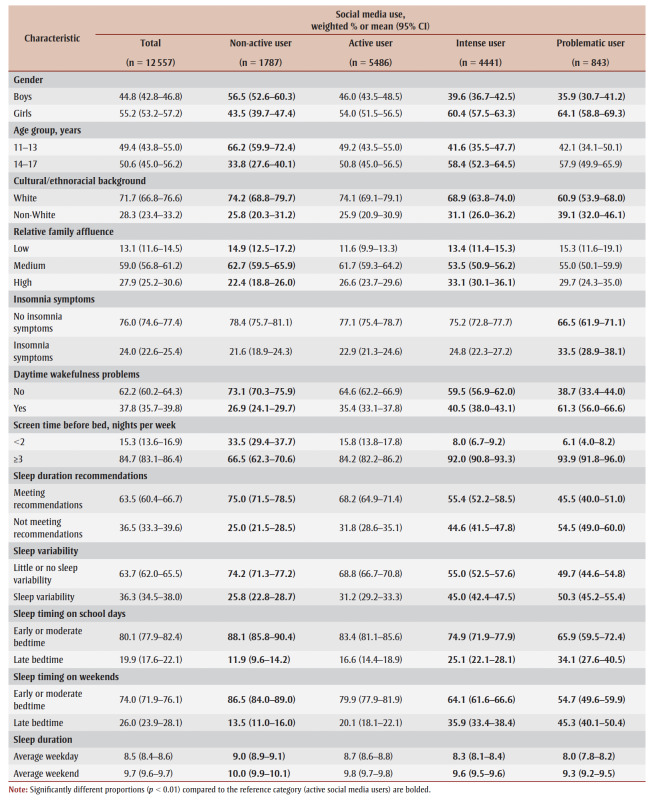

At 9.0 (95% CI: 8.9–9.1) hours per night, 11- to 13-year-olds had a significantly longer mean sleep duration than 14- to 17-year-olds (8.1; 95% CI: 8.0–8.3 hours per night; p<0.001) (data not shown). We also found more 11- to 13-year-olds than 14- to 17-year-olds in the non-active SMU category (66.2% and 33.8%, respectively) compared with the active SMU category (49.2% and 50.8%, respectively) (Table 1).

***Association between SMU and sleep health indicators***

Non-active SMU was associated with significantly lower odds of problematic sleep health indicators compared with active SMU (aORs from 0.42 to 0.78), except for insomnia symptoms, where the association was not significant. Intense SMU was associated with significantly worse sleep for all sleep health indicators except insomnia symptoms (aORs from 1.14 to 2.24). Finally, problematic SMU had the highest odds of having poor sleep health indicators (aORs from 1.67 to 3.24). All of the aORs for problematic SMU and insomnia symptoms, were consistently greater than the aORs for intense SMU, although some not significantly (Table 2).

**Table 2:** Adjusted odds ratios of sleep health indicators by social media use category (n = 12 557)

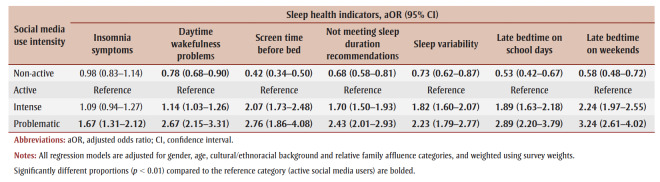

***Stratified analyses***

**Figure 1:**
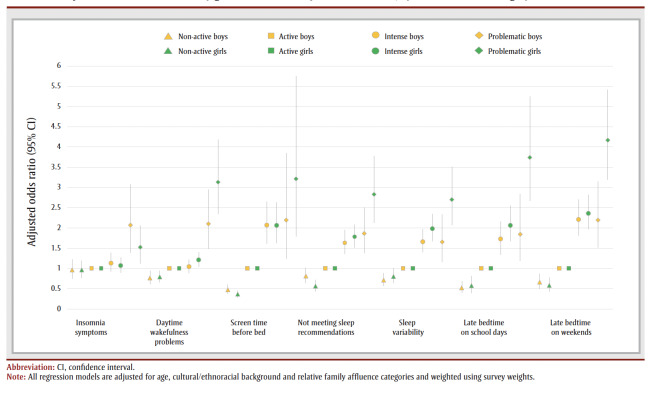
Adjusted odds ratios stratified by gender for seven sleep health indicators, by social media use category (n = 12 557)

The odds of having poor sleep health indicators were greater for problematic users who were girls than for problematic users who were boys, compared to their active user peers; these indicators included insomnia symptoms (aOR = 4.13 and 2.18, respectively), having daytime wakefulness problems (aOR = 3.09 and 2.11, respectively), using screens 3 or more nights a week (aOR = 3.23 and 2.21, respectively), not meeting sleep duration recommendations (aOR = 2.83 and 1.86, respectively), sleep variability (aOR = 2.71 and 1.65, respectively), having a later bedtime on school days (aOR = 3.74 and 1.84, respectively) and having a later bedtime on non-school days (aOR = 4.13 and 2.18, respectively) (Figure 1). 

A similar relationship was found for girl intense users and boy intense users, compared to their active user peers, including insomnia symptoms (aOR = 2.33 and 2.19, respectively), daytime wakefulness problems (aOR = 1.20 and 1.05, respectively), not meeting sleep duration recommendations (aOR = 1.77 and 1.62, respectively), sleep variability (aOR = 1.99 and 1.66, respectively), and having a later bedtime on school days (aOR = 2.06 and 1.72, respectively) and non-school days (aOR= 2.33 and 2.19, respectively). Odds ratios of having poor sleep health indicators did not differ significantly between girl non-active users and boy non-active users compared to their active user peers.

**Figure 2:**
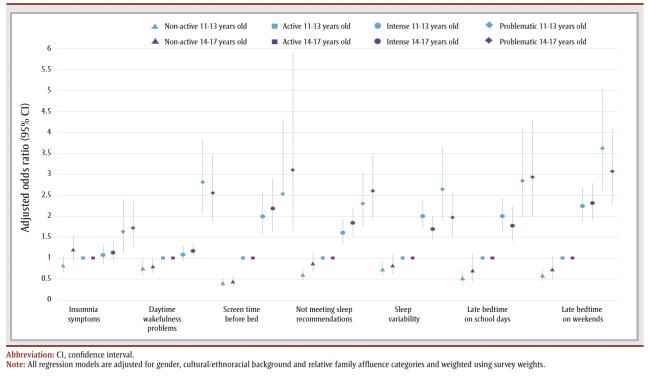
Adjusted odds ratios stratified by age for seven sleep health indicators, by social media use category (n = 12 557)

Overall, odds ratios for having poor sleep health indicators did not differ significantly between the 11- to 13-year-olds and the 14- to 17-year-olds across SMU categories (Figure 2).

## Discussion

In this study, we examined associations between SMU and seven sleep health indicators in a nationally representative sample of adolescents in Canada. Our results show that both problematic and intense SMU were associated with worse sleep health across a range of indicators, compared to active SMU, while non-active SMU was associated with better sleep health. The presence of insomnia symptoms was the only indicator not associated with SMU. Associations were stronger for girls than for boys, but did not differ by age group. The associations for problematic SMU were generally stronger than for intense SMU with all the sleep health indicators. 

Compared to previous research exploring the relationship between SMU and sleep,^12^,^24^-^26^ our study offers a comprehensive examination of the relationship between SMU and different indicators of sleep health. Few studies have made a distinction between intense and problematic SMU and their associations with sleep. Our results point to both the intensity and problematic nature of SMU affecting many aspects of healthy sleep. A few explanations for these associations have been proposed. First, electronic devices (e.g. cellphones, tablets, computer screens) emit blue light, which affects the production of melatonin, a hormone that regulates circadian rhythms and sleep.^6^,^7^ Second, social media activities can lead to psychophysiological arousal, in part because of the emotional content of social media, which can lead to difficulties falling asleep.^14^,^27^ Third, the “never-ending” nature of social media can make it difficult to stop use at night, particularly for adolescents, who are still developing their capacity to self-regulate.^14^,^24^ Fourth, the constant alerts may disrupt the sleep of the large number of adolescents who keep their phones in their bedrooms at night.^14^,^24^ Research has found that 15% of those in France report disturbance of sleep because of text messages alerts.^28^ Further, “fear of missing out,” a general state of anxiety at missing out on experiences, may prevent young people from turning off their phones at night and disengaging from social media at bedtime.^15^ This fear may contribute to psychophysiological activation before bedtime and to delayed bedtimes.^15^

Previous findings on the link between SMU and various health outcomes (e.g. mental health, physical activity) suggest a curvilinear relationship where both non-active and problematic use are associated with health risks relative to active use; this is referred to as the Goldilocks hypothesis.^29^ However, we found that non-active use was associated with better sleep indicators than active use, suggesting a monotonic relationship between SMU and poor sleep health. Notably, intense users had worse sleep health outcomes than non-active and active users, even if their use was not problematic.

We found a stronger association between problematic SMU and sleep indicators among girls than boys. Previous work has shown that, even when using screens for less than 2 hours per day, girls were more likely to experience insufficient sleep than boys.^30^ However, to our knowledge, no study has examined gender differences in intense and problematic SMU and sleep. There is evidence that girls and boys use social media differently and that girls may be more susceptible to the negative impacts of SMU.^14^ For example, girls are more likely to engage in social comparison and seek feedback on social media, which may influence their body image concerns and possibly explains why their sleep is affected to a greater extent.^17^,^31^ They may also be more susceptible to the psychophysiological arousal effects of social media; they report greater emotional investment and increased stress linked to SMU.^26^ A longitudinal study of adolescents in the Netherlands found that social media stress was associated with greater daytime sleepiness among girls but not boys.^26^ Finally, research also suggests a difference between active (e.g. posting or commenting) and passive (e.g. viewing posts, scrolling) SMU. Passive SMU is associated with lower well-being, with a stronger effect in girls,^32^ that might translate into girls spending more time on social media at night and more sleep difficulties.

Overall, we did not find statistically significant differences between 11- to 13-year-olds and 14- to 17-year-olds in associations between SMU and sleep indicators. To the best of our knowledge, our study is among the first to examine age differences in the association between SMU categories and sleep health indicators. Sleep health tends to change with age, with older adolescents sleeping less and going to bed later, partly due to biological changes.^7^,^11^ Time spent on social media also increases as adolescents age, which could explain why the associations between SMU categories and sleep health remain similar with age.

***Strengths and limitations***

There are several strengths to this study. First, it uses nationally representative data, with data collection following a standardized protocol. The study also included a distinction between intense and problematic SMU, using a validated scale for problematic SMU, and examined seven sleep health indicators that offer a broad picture of sleep health.

Some limitations need to be acknowledged. The use of cross-sectional data does not allow for causal inferences. There may have been unmeasured confounding that we were not able to adjust for in our analysis. In addition, the data were self-reported. Some studies suggest that self-reported sleep duration correlates moderately with actigraphy-measured sleep but that self-reports often overestimate sleep duration, which may have introduced some measurement error.^33^ Only 58% of the Canadian HBSC sample had complete data and were included in our analyses. When comparing the characteristics of our included sample against the excluded samples, we found significant differences in gender, age group, culture/ethnoracial background, SMU and screen time before bed, which may indicate a risk of sampling bias in our study impacting the generalizability of our results (data available from the authors on request). Many of the measures used in this study have not been validated, representing an important area of future research. Our measure of SMU does not specify if the use was active (e.g. communicating with friends and creating content) or passive (e.g. scrolling through feeds), and our study could not distinguish between SMU and exposure to screens in general. We also did not have information on the type of device (i.e. phone, tablet, computer) that may play a moderating role in the associations with sleep health. To more precisely understand how SMU affects sleep, further studies should distinguish between active and passive SMU and account for other types of screen use.

## Conclusion

In our study, intense and problematic SMU were associated with worse sleep health compared to active SMU, whereas non-active SMU was linked to better sleep health. These associations were stronger among girls than boys. 

Further research is needed to understand the underlying mechanisms between SMU and sleep and to investigate potentially important gender differences. To guide public health recommendations, further studies could collect data on specific social media activities and use objective measures of sleep and SMU (such as time spent on social media apps). During the COVID-19 pandemic, adolescents increased their time spent on social media.^4^ Considering our findings, it will be important to examine how changes in youth SMU due to the pandemic have impacted sleep health in adolescents.

## Acknowledgements

The HBSC is an international study carried out in collaboration with World Health Organization/EURO. The International Coordinator of the 2018 survey was Dr. Jo Inchley (Glasgow University, Scotland) and the Data Bank Manager was Dr. Oddrun Samdal (University of Bergen, Norway). The Principal Investigators of the Canadian HBSC study are Dr. Wendy Craig (Queen’s University) and Dr. William Pickett (Brock University and Queen’s University), and its national coordinator is Mr. Matthew King (Queen’s University).

## Funding

The Canadian HBSC study is funded in Canada by the Public Health Agency of Canada (6D016-204692/001/SS).

## Conflicts of interest

None to declare.

Justin J. Lang is one of this journal’s Associate Scientific Editors, but has recused himself from the review process for this article.

## Authors’ contributions and statement

FLP: Conceptualization, Formal analysis, Data curation, Writing – Original draft, Writing – Review & editing

JJL: Formal analysis, Data curation, Writing – Original draft, Writing – Review and editing

BM: Data curation, Writing – Review and editing

IS: Data curation, Writing – Review and editing

KCR: Data curation, Writing – Review and editing

SLW: Data curation, Writing – Review and editing

JPC: Data curation, Writing – Review and editing

IJ: Data curation, Writing – Review and editing

MBN: Data curation, Writing – Review and editing

GG: Conceptualization, Data curation, Formal analysis, Writing – Original draft, Writing – Review and editing 

All authors approved the final manuscript.

The content and views expressed in this article are those of the authors and do not necessarily reflect those of the Government of Canada.
